# Quantum Heat Engines with Complex Working Media, Complete Otto Cycles and Heuristics

**DOI:** 10.3390/e23091149

**Published:** 2021-09-01

**Authors:** Ramandeep S. Johal, Venu Mehta

**Affiliations:** Department of Physical Sciences, Indian Institute of Science Education and Research Mohali, Sector 81, S.A.S. Nagar, Manauli PO 140306, Punjab, India; venumehta05@gmail.com

**Keywords:** quantum thermodynamics, quantum heat engine, quantum Otto cycle, heuristics, majorization, XXX spin-model, Otto efficiency, complete Otto cycle

## Abstract

Quantum thermal machines make use of non-classical thermodynamic resources, one of which include interactions between elements of the quantum working medium. In this paper, we examine the performance of a quasi-static quantum Otto engine based on two spins of arbitrary magnitudes subject to an external magnetic field and coupled via an isotropic Heisenberg exchange interaction. It has been shown earlier that the said interaction provides an enhancement of cycle efficiency, with an upper bound that is tighter than the Carnot efficiency. However, the necessary conditions governing engine performance and the relevant upper bound for efficiency are unknown for the general case of arbitrary spin magnitudes. By analyzing extreme case scenarios, we formulate heuristics to infer the necessary conditions for an engine with uncoupled as well as coupled spin model. These conditions lead us to a connection between performance of quantum heat engines and the notion of majorization. Furthermore, the study of complete Otto cycles inherent in the average cycle also yields interesting insights into the average performance.

## 1. Introduction

Thermodynamics originated as an empirical study of steam engines, which blossomed into a framework of exceptional generality and simplicity. Quantum thermodynamics is an emerging research field that aims to extend classical thermodynamics and statistical physics into the quantum realm, offering new challenges and opportunities in the wake of a host of non-classical features. A dominant interest is to understand energy-conversion processes at length scales and temperatures where quantum effects become imperative. Inspired by our enhanced capabilities towards nanoscale design and control, this endeavour is being pursued by scientists from diverse backgrounds, such as statistical physics, quantum information, quantum optics, many-body physics and so on. In order to lay foundations for technological breakthroughs, a variety of fundamental questions are being addressed, ranging from issues of thermalisation of quantum systems to examining the validity of thermodynamic concepts, such as the definitions of work, heat, efficiency and power at the nanoscale. The accord between quantum mechanics and thermodynamics is yet to fully unfold [1,2,3]. Its fundamental implications have inspired numerous proposals for thermal machines based on quantum working media [4,5,6,7,8,9,10,11,12,13,14,15,16,17,18,19,20,21,22,23,24,25,26,27,28,29,30,31,32,33,34,35,36,37,38,39,40,41,42,43,44,45,46,47,48,49,50]. Two major issues which are addressed in such proposals are as follows: What are the performance bounds of heat engines working in quantum regime and what are the thermodynamic properties of these quantum systems which control these bounds? The performance analysis of various quantum analogues of classical heat engines serve as a test bed to study different extensions of thermodynamic ideas in the quantum world. With the recent development of quantum information technology [51,52,53,54] and a number of interesting results, the study of quantum heat engines (QHEs) has drawn much interest. In fact, the past few years witnessed conducive studies exploring how quantum statistics, discreteness of energy levels, quantum adiabaticity, quantum coherence, quantum measurement and entanglement affect the operation of heat engines and cycles in various experimental set-ups, including trapped ions, transmon qubits and more [55,56,57,58,59,60,61,62,63,64,65,66,67,68,69,70,71,72,73,74,75,76,77,78,79,80,81,82,83,84].

Finite time thermodynamic cycles [31,33,85,86,87,88,89,90,91,92,93,94,95,96,97,98] and the study of open quantum systems [99,100,101,102,103,104,105,106] have drawn significant attention in the recent years. These studies aim to arrive at more practical estimates of the performance measures for these machines. However, the importance of quasi-static models of QHEs lies in the fact that they provide a benchmark against which we can compare the behaviour of finite time or more realistic models of heat engines. A range of quantum working substances has been used to model these QHEs. Amongst these, the study of simple, coupled quantum systems [6,18,29,30,107,108,109,110,111,112,113,114,115,116] can yield important insights into the role of quantum interactions in enhancing the performance of model thermal machines. In particular, an upper bound ($\eta_{\mathrm{ub}}$) for quantum Otto efficiency using two coupled spin-1/2 particles has been obtained which is tighter than the Carnot bound ($\eta_{C}$) [6,18]. However, this upper bound was shown to be violated for a spin-1/2 particle coupled with an arbitrary spin [111].

In this paper, we generalise the above model and treat two arbitrary spins which are coupled through XXX interaction. We derive conditions ensuring the operation of Otto engine with an arbitrary spin (uncoupled model) as well as for the coupled model. We are able to analytically show that coupling can enhance thermal efficiency and derive a new spin-dependent upper bound to the Otto efficiency, which generalizes and obtains, as a special case, the previous bound of Reference [6]. The new bound is dependent on the magnitude of the total spin quantum number $s=s_{1}+s_{2}$. Given the arbitrary magnitudes of the spins and complex nature of the energy spectrum, we focus on the worst-case or best-case scenarios (WCS/BCS) to approach the problem. By proceeding in this manner, we observe that WCS implies a certain majorization relation between the canonical probability distributions of the working medium relative to hot and cold reservoirs. Thus, we discover a robust connection between the performance of our thermal machine and the property of majorization.

We also establish the consistency of our model with the second law. As a novel tool, we study complete Otto cycles (COCs) in order to characterise the performance of our engine. For a COC, the working medium starts and ends in the same state. We notice a subtle difference in the sense in which second law may be applied at the COC level compared to the average level. We show that COCs that follow the second law under a certain operation (say as an engine) suggest conditions applicable for the *average* performance of the machine. In this manner, COCs offer a potentially handy tool of estimating parameters for the operating regime of the machine.

It is apparent that as the quantum working medium becomes complex, an exact analysis becomes intractable. This is especially true when the working medium is neither a few-particles system having a simple energy spectrum nor a medium close to thermodynamic limit where some scaling law may aid in mathematical simplicity [117]. Thus, in order to target this intermediate regime, it is pragmatic to formulate heuristics. A significant motivation for our paper is to explore the use of heuristics in view of the complexity of the given problem. Heuristics have been employed in various disciplines such as cognitive science, behavioural economics and computer science to name a few. A heuristic is a rule of thumb providing insights into the behaviour of a system in the face of complexity or uncertainty [118,119,120]. It must be appreciated that the value of a heuristic lies in providing a shortcut method that requires a simpler analysis, thus trading accuracy and completeness for speed.

The paper is organized as follows. In Section 2, we introduce our model of two coupled spins ($s_{1},s_{2}$) as the working substance of the Quantum Otto engine. In Section 2.1, various stages of the heat cycle are described, and the positive work condition for the uncoupled model is discussed. The proof for the same is sketched in Appendix Appendix A. In Section 3, the spins are coupled, and we find the coupling range in which positive work extraction is ensured (proofs are sketched in Appendix B and Appendix C), which is related to the notion of majorization in Section 3.1 and further used to order the system’s energy levels for J≠0 in Section 3.2. In Section 4, the conditions for maximum enhancement of coupled system’s efficiency over the uncoupled model are discussed. An upper bound to engine’s efficiency is also calculated in the considered domain of coupling. A detailed proof for the positive entropy production for the coupled system is sketched in Appendix D. In Section 5, an analysis is carried out by using the notion of complete Otto cycles. Finally, we discuss the results of our analysis in Section 6.

## 2. Quantum Otto Cycle

The working substance consists of two spins with arbitrary magnitudes, $s_{1}$ and $s_{2}$, coupled by 1-D isotropic Heisenberg exchange interaction in the presence of an externally applied magnetic field of magnitude *B* along the *z*-axis. The system Hamiltonian in the first Stage of the cycle can be written as follows:

(1) $$H_{1}\equiv H_{1}+H_{int}=2B_{1}\left(s_{1}^{\left(z\right)}⊗I+I⊗s_{2}^{\left(z\right)}\right)+8J{\vec{s}}_{1}.{\vec{s}}_{2}$$

where J>0 is the strength of the anti-ferromagnetic coupling. ${\vec{s}}_{1}\equiv \left\{s_{1}^{\left(x\right)},s_{1}^{\left(y\right)},s_{1}^{\left(z\right)}\right\}$ and ${\vec{s}}_{2}\equiv \left\{s_{2}^{\left(x\right)},s_{2}^{\left(y\right)},s_{2}^{\left(z\right)}\right\}$ are the spin operators for the first and the second spin, respectively. $H_{int}$ is the interaction Hamiltonian, and $H_{1}$ is the free Hamiltonian. We have taken Bohr magneton $\mu_{B}=1$, and the gyromagnetic ratio for both spins has been taken to be two [121].

Let *n = $\left(2s_{1}+1\right)\left(2s_{2}+1\right)$* be the total number of energy levels with $|\psi_{k}\rangle$ as the corresponding energy eigenstates. When the system is in thermodynamic equilibrium with a heat bath at temperature *T*, the density matrix $\rho_{1}$ for the working substance can be written as follows:

(2) $$\rho_{1}=\sum_{k=1}^{n}P_{k}|\psi_{k}\rangle \langle \psi_{k}|,$$

where $P_{k}=e^{-E_{k}/T}/Z$ are the occupation probabilities of the energy levels, and $Z=\sum_{k}e^{-E_{k}/T}$ is the partition function for the system. We have rendered the Boltzmann constant $k_{B}$ equal to unity.

**Figure 1:**
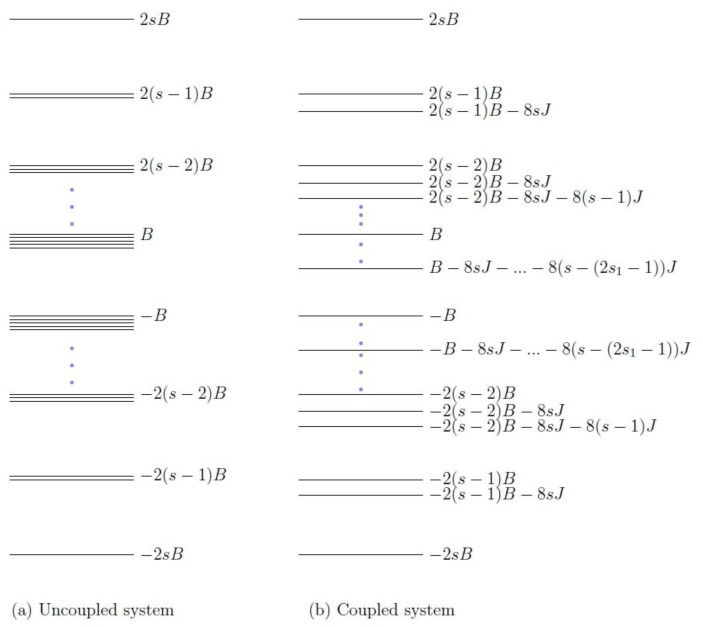
The above figure shows the energy levels of the two-spins ($s_{1},s_{2}$) system for (**a**) J=0 and (**b**) J>0. The degeneracy in the energy levels is lifted as the interaction is switched on (J≠0). Here, $s_{1}<s_{2}$ and $s=s_{1}+s_{2}$.

Let us consider the case where one spin is an integer, and the other is a half integer. Some examples of such spin combinations are $\left(\frac{3}{2},2\right),\left(\frac{1}{2},2\right)$ and $\left(\frac{5}{2},4\right)$. The energy eigenvalues of the Hamiltonian H for a general ($s_{1},s_{2}$) coupling are shown in Figure 1. It is to be noted that a term $8s_{1}s_{2}J$ common in all the eigenvalues has been neglected as the physical properties of the system would be independent of it. The ordering of these energy levels would depend upon the conditions on the parameters which the positive work condition for the system would provide, which will be discussed in the coming sections.

### 2.1. The Heat Cycle

The four stages constituting the Otto cycle are as follows.

*Stage 1:*  The system is at thermal equilibrium with a heat reservoir at temperature $T_{1}$ with energy $e_{k}$ for which its occupation probabilities are $p_{k}$, and the corresponding density matrix is $\rho_{1}$ (here, we are considering two non interacting spins with energy eigenvalues denoted by $e_{k}$ and occupation probabilities by $p_{k}$).
*Stage 2:*  The system undergoes a quantum adiabatic process after it is isolated from the hot bath, and the magnetic field is changed from $B_{1}$ to a smaller value $B_{2}$. Here, the quantum adiabatic theorem is assumed to hold according to which the process should be slow enough so that no transitions are induced as the energy levels change from $e_{k}$ to $e_{k}^{{}^{\prime }}$.
*Stage 3:*  Here, the system is brought in contact with a cold bath at temperature $T_{2}$ (<$T_{1}$). The energy eigenvalues remain at $e_{k}^{{}^{\prime }}$, and the occupation probabilities change from $p_{k}$ to $p_{k}^{{}^{\prime }}$ with the external magnetic field at $B=B_{2}$, and the density matrix of the system is $\rho_{2}$.
*Stage 4:*  The system is detached from the cold bath, and the magnetic field is changed from $B_{2}$ to $B_{1}$ with occupation probabilities remaining unchanged at $p_{k}^{{}^{\prime }}$ and energy eigenvalues changing back from $e_{k}^{{}^{\prime }}$ to $e_{k}$ such that only work is performed on the system during this step. Finally, the system is attached to the hot bath again, and the cycle is completed such that the average heat absorbed is $q_{1,av}=\mathrm{Tr}\left[H_{1}\Delta \rho_{}\right]$, and the net work performed per cycle is $w_{av}=\mathrm{Tr}\left[\left(H_{1}-H_{2}\right)\Delta \rho_{}\right]$. Here, Tr[·] denotes the trace operation, and $\Delta \rho_{}=\rho_{1}-\rho_{2}$. In this paper, we consider the free Hamiltonian of the form $H_{i}\equiv 2B_{i}h_{0}$ (i=1,2), where $h_{0}$ is an operator. We now have $w_{av}=2\left(B_{1}-B_{2}\right)\mathrm{Tr}\left[h_{0}\Delta \rho_{}\right]$; therefore, the efficiency in the absence of interaction is as follows.
  (3) $$\eta_{0}=1-\frac{B_{2}}{B_{1}}.$$

Let us first discuss the positive work condition when $s_{1}$ and $s_{2}$ are non-interacting. The energy eigenvalues ($e_{k}$) of the free Hamiltonian, written in the order of increasing energy (if one spin is integer and the other is half integer), are listed in Table 1, and as it can be observed many energy levels for the non-interacting system are degenerate. There is only one level with energy proportional to −s as well as *s*, two levels with energy proportional to −(s−1) as well as (s−1) and so on (the proportionality constant always being 2B). Therefore, denoting the degeneracy by “g”, we have the following from Table 1:

(4) $$g_{\left|s\right|}=1,\;g_{\left|s-1\right|}=2,\;g_{\left|s-2\right|}=3,...,g_{\left|s-r\right|}=2s_{1}+1$$

such that the total number of energy levels are as follows.

$$n=\left(2s_{1}+1\right)\left(2s_{2}+1\right)=2\left(g_{\left|s\right|}+g_{\left|s-1\right|}+g_{\left|s-2\right|}+...+g_{\left|s-r\right|}\right).$$

The *Stage 1* occupation probabilities are written as $p_{k}=e^{-e_{k}/T_{1}}/z_{1}$, where $z_{1}=\sum_{k=1}^{n}e^{-e_{k}/T_{1}}$ is the partition function of the system, which can be expressed as follows.

(5) $$z_{1}=2\sum_{l=1}^{s+1/2}g_{\left|s-l+1\right|}.\cosh\left[2\left(s-l+1\right)B_{1}/T_{1}\right].$$

The average heat exchanged with the hot reservoir is as follows:

(6) $$q_{1,av}=\sum_{k=1}^{n}e_{k}\left(p_{k}-p_{k}^{{}^{\prime }}\right)=2B_{1}v,$$

where the primed probabilities are tabulated at $T=T_{2}$ and $B=B_{2}$. The average heat exchanged with the cold bath is as follows.

(7) $$q_{2,av}=\sum_{k=1}^{n}e_{k}^{{}^{\prime }}\left(p_{k}^{}-p_{k}^{{}^{\prime }}\right)=2B_{2}v,$$

Thus, the work performed on average is the following.

(8) $$w_{av}=q_{1,av}-q_{2,av}=2\left(B_{1}-B_{2}\right)v.$$

The explicit expression of *v* is given by Equation (A1). Since $B_{1}>B_{2}$ is assumed, the system works as an engine on average if and only if v>0. We prove in Appendix A that the condition required to satisfy v>0 is the following:

(9) $$\frac{B_{2}}{T_{2}}>\frac{B_{1}}{T_{1}},\;\;\mathrm{or}\;\;B_{2}>B_{1}\theta ,$$

where $\theta =T_{2}/T_{1}$. Furthermore, as proved in Appendix A, Equation (9) implies $z_{2}>z_{1}$ as well as the following.

(10) $$p_{1}^{{}^{\prime }}>p_{1},\;\mathrm{and}\;p_{n}^{{}^{\prime }}<p_{n},$$

From the above conditions, we can make the following inferences. Positive work extraction is favoured when the occupancy of ground (top) level is more (less) at the cold bath than at the hot bath, which suggests that heat is absorbed at the hot bath, decreasing (increasing) the occupancy of the ground (top) level, while heat is released at the cold bath, thus increasing (decreasing) the occupancy of the ground (top) level.

Since the working medium returns to its initial state (restoring the Hamiltonian as well as coming to be in equilibrium with the hot reservoir), the net change in entropy $\Delta S_{0,av}$ is due to the entropy changes only in the heat baths. The decrease in the entropy of the hot bath is $-q_{1,av}/T_{1}$ and increase in entropy of the cold bath is $q_{2,av}/T_{2}$. Thus, the net entropy change in one cycle is the following.

(11) $$\Delta S_{0,av}=-\frac{q_{1,av}}{T_{1}}+\frac{q_{2,av}}{T_{2}}=\left(-\frac{B_{1}}{T_{1}}+\frac{B_{2}}{T_{2}}\right)v.$$

We have seen that $w_{av}>0$ or v>0 requires Equation (9) to hold. Under these conditions, it follows that $\Delta S_{0,av}>0$, and the consistency with the second law is established at the level of average performance as an engine. Similarly, we observe that the efficiency satisfies the following. $\eta_{0}<1-T_{2}/T_{1}=\eta_{C}$.

## 3. The Coupled Model

Let us now couple the two spins, with J>0 being the anti-ferromagnetic coupling strength. The corresponding energy eigenvalues are shown in Figure 1b, where the ordering of the eigenvalues can be considered when the coupling parameter *J* is small. Moreover, as the coupling is switched on, the degeneracy of the previously degenerate levels is now lifted. Let us express an energy eigenvalue of the coupled system as $E_{k}=m_{1}B-8m_{2}J$, where $m_{1}=-2s,...,+2s$ and $m_{2}$ can only take positive values including zero, as shown in Table A2 in Appendix C. The values $m_{1}$ and $m_{2}$ depend on the index *k*, but we have omitted it here for brevity of notation.

Now, the average heat absorbed from the hot bath ($Q_{1,av}$), the heat rejected to the cold bath ($Q_{2,av}$) and the average work performed in one cycle, $W_{av}=Q_{1,av}-Q_{2,av}$, are given as follows:

$$\begin{array}{ccc} Q_{1,av} & = & 2B_{1}X+8JY,\\ Q_{2,av} & = & 2B_{2}X+8JY,\\ W_{av} & = & 2(B_{1}-B_{2})X, \end{array}$$

where the following is the case.

(12) $$X=\frac{1}{2}\sum_{k=1}^{n}m_{1}\left(P_{k}^{}-P_{k}^{{}^{\prime }}\right),\;Y=\sum_{k=2}^{n-2}m_{2}\left(P_{k}^{{}^{\prime }}-P_{k}\right).$$

The spin dependent factors $m_{1}$ and $m_{2}$ are obtained from the expressions of the equilibrium occupation probabilities of the energy levels $E_{k}$ (shown in Figure 1), which in general are written as follows.

(13) $$P_{k}=\frac{e^{-m_{1}B_{1}/T_{1}+8m_{2}J/T_{1}}}{Z_{1}}.$$

For explicit expressions of $P_{k}$, refer to Table A1 in Appendix B. $Z_{1}$ is the *Stage 1* partition function of the system for which its expression may be rewritten as follows:

(14) $$Z_{1}=\left\{\begin{array}{c} Z_{1}+2\cosh\left[2\left(s-1\right)B_{1}/T_{1}\right].e^{8sJ/T_{1}}+\\ 2\cosh\left[2\left(s-2\right)B_{1}/T_{1}\right].\left(e^{8sJ/T_{1}}+e^{8\left(s-1\right)J/T_{1}}\right)+...+\\ 2\cosh\left[2\left(s-r\right)B_{1}/T_{1}\right].\left(e^{8sJ/T_{1}}+e^{8\left(s-1\right)J/T_{1}}+...+e^{8\left(s-\left(2s_{1}-1\right)\right)J/T_{1}}\right), \end{array}\right.$$

where $Z_{1}\equiv 2\sum_{k=1}^{s+1/2}\cosh\left[2\left(s-k+1\right)B_{1}/T_{1}\right]$. Similarly, we can define $P_{k}^{{}^{\prime }}$, the canonical probabilities due to cold bath, by replacing $B_{1}\to B_{2}$ and $T_{1}\to T_{2}$ in the above expressions for $P_{k}^{}$.

For the proof of PWC for the coupled model (Appendix B), we show that for the so-called worst case scenario (WCS) is given by the following:

(15) $$P_{k}^{{}^{\prime }}<P_{k}^{},\;k=2,3,...,n,\;\mathrm{and}\;P_{1}^{{}^{\prime }}>P_{1},$$

along with Equation (9). It follows that X>0. Consistent with Equations (15) and (A25), we then calculate the strictest condition on the allowed range of *J* (Appendix C), which is given by the following.

(16) $$0<J<\frac{B_{2}-B_{1}\theta }{4s\left(1-\theta \right)}\equiv J_{c}.$$

Therefore, we conclude that X>0 or PWC is satisfied under Equations (9) and (16), with the latter constituting the sufficient condition for the coupled system to work as an engine.

### 3.1. Majorization

Majorization [122] is a powerful mathematical concept that defines a preorder on the vectors of real numbers. It is particularly useful to compare two probability distributions. We will highlight its occurance in the context of the working regime of our engine by comparing the two equilibrium probability distributions.

Now, for the uncoupled model, the relevant probability distributions are the canonical probabilities $\left\{p_{k}\right\}$ and $\left\{p_{k}^{{}^{\prime }}\right\}$, which, at finite temperatures, are ordered as $p_{n}^{}<p_{n-1}^{}<\cdots <p_{1}^{}$ and $p_{n}^{{}^{\prime }}<p_{n-1}^{{}^{\prime }}<\cdots <p_{1}^{{}^{\prime }}$, respectively. In Lemma A2 of Appendix B, we proved that Equation (9) is a necessary condition that ensures $w_{av}>0$ in the regime of the so-called worst case scenario (WCS), given by the following:

$$p_{k}^{{}^{\prime }}\leq p_{k}^{},\;k=2,3,...,n\;\mathrm{and}\;p_{1}^{{}^{\prime }}\geq p_{1}^{},$$

where the equality holds for $B_{2}/T_{2}=B_{1}/T_{1}$. Therefore, the above relations imply the following.

$$\begin{array}{cc} p_{n}^{{}^{\prime }} & \leq p_{n},\\ p_{n}^{{}^{\prime }}+p_{n-1}^{{}^{\prime }} & \leq p_{n}^{}+p_{n-1}^{},\\ \; & \vdots \\ \sum_{k=1}^{n-1}p_{k}^{{}^{\prime }} & \leq \sum_{k=1}^{n-1}p_{k},\\ \sum_{k=1}^{n}p_{k}^{{}^{\prime }} & =\sum_{k=1}^{n}p_{k}. \end{array}$$

The above set of conditions (M) is summarised by stating that $\left\{p_{k}^{{}^{\prime }}\right\}$ *majorizes* $\left\{p_{k}^{}\right\}$ and denoted as $\left\{p_{k}\right\}≺\left\{p_{k}^{{}^{\prime }}\right\}$. As a powerful tool, majorization can be used to prove other results. Intuitively, it indicates that the distribution $\left\{p_{k}^{}\right\}$ is more mixed than $\left\{p_{k}^{{}^{\prime }}\right\}$. Thus, as an important consequence, $\left\{p_{k}\right\}≺\left\{p_{k}^{{}^{\prime }}\right\}$ implies that $S\left(p_{k}\right)\geq S\left(p_{k}^{{}^{\prime }}\right)$, where S(p) is the Shannon entropy of the distribution {p} (proportional to the thermodynamic entropy of the working medium in equilibrium with a reservoir). In fact, this is expected, since the flow of heat for the engine is on the average from hot to cold. Then, along with heat, thermodynamic entropy is also lost to the cold reservoir. However, the condition of majorization is more general than the above mentioned relation between the entropies.

Similarly for the coupled model, we have shown that Equations (9) and (16) ensure $W_{av}>0$ under the following conditions: $P_{k}^{{}^{\prime }}<P_{k}^{},\forall \;k=2,3,...,n$ and $P_{1}^{{}^{\prime }}>P_{1}^{}$. In general, we may write the following.

(17) $$P_{k}^{{}^{\prime }}\leq P_{k}^{};\;k=2,3,...,n\;\mathrm{and}\;P_{1}^{{}^{\prime }}\geq P_{1}^{}.$$

Thus, for the coupled model, we can also write down the set of conditions equivalent to Equation (M), and infer that $\left\{P_{k}\right\}≺\left\{P_{k}^{{}^{\prime }}\right\}$, which implies $S\left(P_{k}\right)\geq S\left(P_{k}^{{}^{\prime }}\right)$. In other words, if the *Stage 3* equilibrium distribution majorizes *Stage 1* equilibrium distribution, then we have positive work extraction from the coupled system.

**Figure 2:**
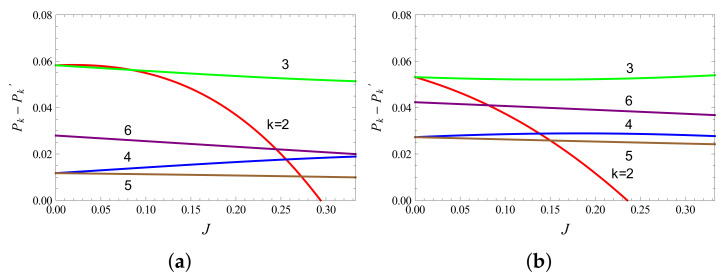
(**a**) Variation of $P_{k}^{}-P_{k}^{{}^{\prime }}$ with the coupling factor *J* for (1/2,1) system, with *k* values ranging from 2 to 6. The parameters are set at $B_{1}=4,B_{2}=3$, with temperatures (**a**) $T_{1}=4,T_{2}=2$ and (**b**) $T_{1}=6,T_{2}=3$. Here, $J_{c}=1/3$. The value of *J* for which $P_{2}^{}-P_{2}^{{}^{\prime }}$ (red curve) changes sign (from positive to negative) approaches $J_{c}$ for lower temperatures (see also Figure 3).

**Figure 3:**
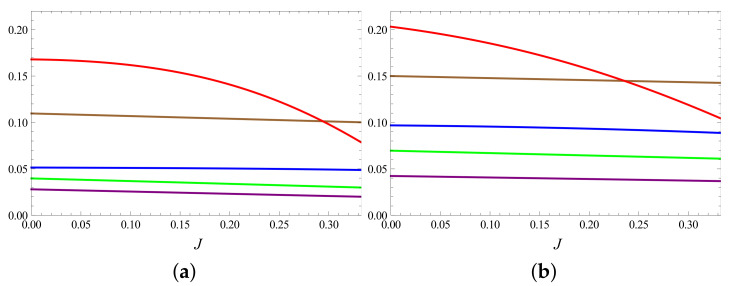
Majorization conditions shown by positivity of all quantities $P_{6}^{}-P_{6}^{{}^{\prime }}$ (purple), $\sum_{k=5}^{6}P_{k}^{}-P_{k}^{{}^{\prime }}$ (green), $\sum_{k=4}^{6}P_{k}^{}-P_{k}^{{}^{\prime }}$ (blue), $\sum_{k=3}^{6}P_{k}^{}-P_{k}^{{}^{\prime }}$ (brown) and $\sum_{k=2}^{6}P_{k}^{}-P_{k}^{{}^{\prime }}$ (red) as function of the coupling strength *J* for (1/2,1) system of Figure 2. The parameters are set at $B_{1}=4,B_{2}=3$, with temperatures (**a**) $T_{1}=4,T_{2}=2$ and (**b**) $T_{1}=6,T_{2}=3$. The point where the red curve intersects the lower curve is where $P_{2}^{{}^{\prime }}=P_{2}$. It is observed that for higher bath temperatures (for a given ratio $T_{2}/T_{1}$), this point shifts to lower *J* values.

It is possible to find a range of parameter values which satisfy Equation (17). In Figure 2, we show the behaviour of $\left(P_{k}^{}-P_{k}^{{}^{\prime }}\right)$ for (1/2,1) system. It is observed that $\left(P_{2}-P_{2}^{{}^{\prime }}\right)$ changes sign within the range $\left[0,J_{c}\right]$, indicating that every condition of Equation (17) may not hold in this range, especially at high bath temperatures. However, we observe that the majorization conditions continue to hold and $\left\{P_{k}\right\}≺\left\{P_{k}^{{}^{\prime }}\right\}$, even if $P_{2}^{{}^{\prime }}>P_{2}$ (see Figure 3).

### 3.2. Energy Level Ordering

The actual arrangement of the energy eigenvalues depends on the positive work conditions derived above. As for the relative position of 2sB energy level, it will not change, because it is the highest energy eigenvalue of the system regardless of the coupling strength *J*. The ground state or the minimum energy state will be decided as follows.

There are two energy levels −2sB and −2(s−1)B−8sJ which can possibly form the ground state of the coupled system, and their energy gap is |2B−8sJ|. Given that $B_{1}>B_{2}$ and $0<J<J_{c}$, we can check that the following is the case.

(18) $$J<J_{c}<\frac{B_{2}}{4s}<\frac{B_{1}}{4s}.$$

The above implies that 2B−8sJ>0, thereby making −2sB the lowest energy of the system and −2(s−1)B−8sJ the energy of the first excited state. Now, Equation (18) opens different possibilities for the arrangement of other energy levels. For example, the levels −2(s−2)B−8sJ−8(s−1)J and −2(s−1)B have an energy gap of |−2B+8sJ+8(s−1)J|, and either of them can be at higher energy state than the other, and both the arrangements are acceptable. For the sake of concreteness, we assume the condition that there is no level crossing when $B_{1}$ is changed to a lower value $B_{2}$. One method of arranging the energy levels, in accordance with Equation (18), is shown in Figure 1, which is assumed for the discussion that follows. The net entropy production in one cycle $\Delta S_{av}$ for the coupled system $\Delta S_{av}=-Q_{1,av}/T_{1}+Q_{2,av}/T_{2}$ can be written as follows.

(19) $$\Delta S_{av}=2X\left(\frac{B_{2}}{T_{2}}-\frac{B_{1}}{T_{1}}\right)+8JY\left(\frac{1}{T_{2}}-\frac{1}{T_{1}}\right).$$

In the above expression, due to Equation (9), the first term is always positive, but since $T_{1}>T_{2}$, the sign of the second term depends on *Y* which may not be positive. We will consider the WCS whereby under Equation (15), all terms in the defining sum *Y* (Equation (12)) are negative, thus making *Y* negative definite (note that $m_{2}>0$ for all *k*). By defining the following:

$$Y_{1}=-Y/s,\;a=2\left(\frac{B_{2}}{T_{2}}-\frac{B_{1}}{T_{1}}\right)>0\;b=8sJ\left(\frac{1}{T_{2}}-\frac{1}{T_{1}}\right)>0,$$

we have $\Delta S_{av}=aX-bY_{1}$. The condition, given by Equation (16), on the coupling strength which ensures $W_{av}>0$ implies that a>b. Then, for $Y_{1}>0$, we have shown in Appendix D that PWC for the coupled system encapsulated in Equations (9) and (16) suffice to prove $X>Y_{1}$ and hence $\Delta S_{av}>0$. This establishes the consistency of our engine with the second law in the considered domain.

## 4. Efficiency Enhancement and the Upper Bound

In the above, we have established conditions for work extraction in the quantum Otto cycle for the coupled system and verified consistency with the second law. In this section, we explore how the coupling between the spins may enhance the efficiency of the engine.

The heat absorbed from the hot reservoir is given by $Q_{1,av}=2B_{1}X+8JY$, where *X* and *Y* are as defined in Equation (12). From the energy levels diagram, it is clear that the contribution 8JY to the exchanged heat comes solely from levels which depend on parameter *J* apart from the field *B*. Now, since $Q_{2,av}=2B_{2}X+8JY$, this ’extra’ contribution to heat is not available for conversion into work, and it is wasted if 8JY>0. However, it may be utilized to enhance the efficiency of the cycle if 8JY<0, thus effectively decreasing the heat absorbed from the hot reservoir. Remarkably, the WCS considered earlier implies that all terms entering the sum for *Y* are negative, and so we have Y≤0 with J>0. Thus, the WCS directly results in a regime where we can expect an enhancement of the efficiency. Thus, for the operational regime discussed in previous sections, we can rewrite the expression for efficiency, $\eta =1-Q_{2,av}/Q_{1,av}$ as follows:

(20) $$\eta =\frac{\eta_{0}}{1+\frac{8JY}{2XB_{1}}}=\frac{\eta_{0}}{1-\frac{4sJY_{1}}{XB_{1}}}$$

where $Y_{1}=-Y/s>0$. We have proved in Appendix D that $X>Y_{1}$. With $B_{1}>4sJ$ (Equation (18)), we obtain the following:

(21) $$\eta <\frac{\eta_{0}}{1-4sJ/B_{1}}<1-\frac{T_{2}}{T_{1}}=\eta_{C},$$

where the second inequality follows due to the permissible range of *J* (Equation (16)). Thus, the expression of the following:

(22) $$\eta_{ub}=\frac{\eta_{0}}{1-4sJ/B_{1}}$$

constitutes an upper bound to the system’s efficiency, which is tighter than the Carnot efficiency, and is within the coupling range $0<J<J_{c}$.

The above expression bounding the efficiency of the Otto cycle is our main result of the paper. This expression is validated with numerical calculations in the discussion section. Note that $\eta_{ub}$ given by Equation (22) is dependent solely on the field values and the total spin of the two particles, while it is independent of the bath temperatures. This expression generalizes the upper bound derived earlier in Reference [6] for the $\left(\frac{1}{2},\frac{1}{2}\right)$ system.

We close this section with a remark on the three possible spin combinations for our ($s_{1},s_{2}$) system:

- When one spin value is a half-integer and the other is an integer;
- When both values are half-integer or both are integers;
- When both are of the same magnitude (both as half-integer or integer).

In this paper, we have discussed the first case only. The only difference between the present case and the other two cases is that, for the latter, when the spins are uncoupled, an energy level with zero energy and $2s_{1}+1$-fold degeneracy occurs but that does not affect the performance of the system. The reason is that after the coupling is turned on between the spins, this energy state splits into $2s_{1}+1$ non-degenerate energy levels, which depend only on the coupling factor *J*. Since *J* is kept fixed during the cycle, these levels do not shift in a cycle and hence do not contribute to the average work resulting in the same PWC as already derived for the first case. Similarly, it can be observed that these levels do not change the condition for maximal efficiency enhancement, and same upper bound can be obtained whatever the spin combination may be.

## 5. Complete Otto Cycles

The working medium for the classical Otto cycle is usually a macroscopic system amenable to thermodynamic treatment. This medium may be a collection of statistically independent, non-interacting individual quantum systems or *elements*, such as spin-1/2 particles or harmonic oscillators and so on. In the adiabatic step of the Otto cycle, the thermodynamic entropy of the working medium stays constant. This implies that there is no intrinsic control on the transitions experienced by individual elements of the working medium.

On the other hand, the working medium of a quantum Otto engine consists of individual elements. In a quasi-static cycle, the isochoric steps are stochastic while the adiabatic steps are deterministic. The quantum adiabatic step is executed slowly enough such that no transition is induced between energy levels of the element, which continues to occupy its initial state throughout the process. Thus, at the level of the ensemble, the occupation probabilities do not change during this process. Thus, such a process imposes maximal control on the evolution of the isolated element, and it is described by a quantum unitary process.

Still, due to the stochastic nature of the contact with the reservoirs, the element may not return to its initial state after the four steps of the cycle. Usually, we are interested in the average properties of the cycle by which the quantities such as heat and work are defined at the ensemble level. In this section, we focus on the complete Otto cycles (COCs) inherent in the average Otto cycle considered in earlier sections. The reason that Otto cycle is so often studied in the quantum thermodynamics literature is that the contributions towards heat and work can be clearly separated into different steps, which helps in the analysis. This distinction also holds at the level of COCs; the interaction of the working medium with a reservoir involves only the exchange of heat with the reservoir, whereas the quantum adiabatic step involves only work.

**Figure 4:**
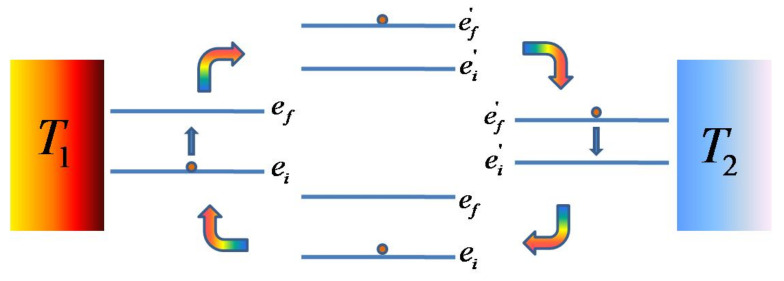
Schematic of a complete Otto cycle (COC) as an engine using two heat reservoirs ($T_{1}>T_{2}$), involving two energy levels of the working medium. The heat absorbed from the hot reservoir is $q_{1}=e_{f}-e_{i}$, while the heat rejected to the cold bath is $q_{2}=e_{f}^{{}^{\prime }}-e_{i}^{{}^{\prime }}$. The work extracted per complete cycle is $w=q_{1}-q_{2}$.

Consider the COC shown as an engine in Figure 4. If the working medium starts at energy level $e_{i}$, then by the end of the four stages it is again found at level $e_{i}$. Such a cycle can either run forward as an engine or backwards as a refrigerator. Analysing the performance of COCs is much easier since we are dealing with only two levels at a time without invoking occupation probabilities of the levels and any average quantities.

Let us represent an energy eigenvalue of the uncoupled system as $e\equiv m_{1}B$, where $m_{1}$ varies from $m_{1}=-2s,...,+2s$. Based on the final (*f*) and initial (*i*) values of $m_{1}$, let us define the quantity $x=m_{1,f}-m_{1,i}$, ranging as x=±2,...,±4s. Let $q_{1},q_{2},w$ denote the heat exchanged with the hot bath, cold bath and the work performed, respectively.

$$\begin{array}{ccc} q_{1} & = & e_{f}-e_{i}=xB_{1},\\ q_{2} & = & e_{f}^{{}^{\prime }}-e_{i}^{{}^{\prime }}=xB_{2},\\ w & = & q_{1}-q_{2}=x\left(B_{1}-B_{2}\right). \end{array}$$

With $B_{1}>B_{2}>0$, we have $q_{h},q_{c}>0$ and w>0 if x>0. It is clear that for x>0 (x<0), a COC runs as an engine (refrigerator). The net entropy change ($\Delta S_{0}$) is contributed only by the reservoirs. Thereby, we obtain the following.

(23) $$\Delta S_{0}=-\frac{q_{1}}{T_{1}}+\frac{q_{2}}{T_{2}}=x\left(-\frac{B_{1}}{T_{1}}+\frac{B_{2}}{T_{2}}\right).$$

Now, for x>0, the condition $B_{2}/T_{2}>B_{1}/T_{1}$ ensures that $\Delta S_{0}>0$, or we may say that the second law is then satisfied at the level of COC. Note that there is a subtle difference in the statement about the second law at the level of a COC versus the average performance level. In the former case, x>0 guarantees the operation of an engine, whereas the *additional* condition (9), i.e., $B_{2}/T_{2}>B_{1}/T_{1}$ makes this operation consistent with the second law. On the other hand, for the average operation as an engine, we require v>0, which itself requires the condition (9). The latter then automatically ensures consistency with the second law at the level of average performance.

Moreover, note that we do not impose the second law at the level of a COC, and the net entropy change for a COC may be negative, as for instance with x<0 or a COC operating as a refrigerator if $B_{2}/T_{2}>B_{1}/T_{1}$. Thus, we do not imply that COCs with $\Delta S_{0}<0$ do not happen. These observations result in the following interesting conclusion about the uncoupled model. *A consistency with the second law for the average performance as engine ensures consistency with the second law for a COC as engine and vice versa*.

Let us next study the effect of coupling between the spins. Now, there are no degenerate levels. Let us express an energy eigenvalue of the coupled system as $E\equiv m_{1}B-8m_{2}J$, where the $m_{1},m_{2}$ values are given in Table A2. The levels with same $m_{1}$ were originally degenerate in the uncoupled model. For the coupled model, energy levels belong to the same band if they have the same value of $m_{1}$ but have different values of $m_{2}$. Furthermore, note that in every band there is one level that stays at the same energy even after the coupling is switched on. Now, for a COC between any two energy levels of the coupled system, the general forms of heat exchanged with the reservoirs, $Q_{1}$ and $Q_{2}$ and the work performed, $W=Q_{1}-Q_{2}$, can be written as follows:

$$\begin{array}{ccc} Q_{1} & = & xB_{1}+8Jy,\\ Q_{2} & = & xB_{2}+8Jy,\\ W & = & x(B_{1}-B_{2}), \end{array}$$

with $x=m_{1,f}-m_{1,i}$ and $y=m_{2,f}-m_{2,i}$. The net entropy change in one cycle is the following.

(24) $$\Delta S=-\frac{Q_{1}}{T_{1}}+\frac{Q_{2}}{T_{2}}=x\left(\frac{B_{2}}{T_{2}}-\frac{B_{1}}{T_{1}}\right)+8Jy\left(\frac{1}{T_{2}}-\frac{1}{T_{1}}\right).$$

We discuss the possible COCs as below stated below.

1. x≠0,y=0: These cycles occur between any two different energy bands having the same $m_{2}$. Therefore, if such a cycle proceeds as an engine (x>0), its efficiency is $W/Q_{1}=1-B_{2}/B_{1}=\eta_{0}$. From Equation (24), this COC is consistent with the second law for $B_{2}>B_{1}\theta$.
2. x=0,y≠0: These cycles are possible between energy levels of the same band, i.e., having same $m_{1}$. The work performed is zero, and the heat exchanged is $Q_{1}=8Jy=Q_{2}$. Thus, for y>0, the corresponding efficiency is also zero.
3. x,y≠0**with the same sign**: These cycles are possible between different bands for levels with different $m_{1}$ and $m_{2}$. If such cycles proceed as engine, i.e., x>0 (and y>0), then the corresponding efficiency is the following.
  (25) $$\eta_{}=\frac{\eta_{0}}{1+\frac{8yJ}{xB_{1}}}<\eta_{0}.$$
  From Equation (24), this type of COC is consistent with the second law for $B_{2}>B_{1}\theta$, *without* imposing any further condition on the coupling strength J≥0. Therefore, if the second law allows COCs with $\eta =\eta_{0}$, then it also allows COCs with $\eta <\eta_{0}$.
4. x,y≠0**with opposite signs**: These cycles occur between energy levels of different bands with different $m_{1}$ and $m_{2}$. If x>0 for such cycles (and y<0), the corresponding efficiency is as follows.
  (26) $$\eta =\frac{\eta_{0}}{1-\frac{8|y|J}{xB_{1}}}>\eta_{0}.$$

From Equation (24), this COC is allowed by the second law if $B_{2}>B_{1}\theta$ and the following is the case.

(27) $$0<J<\frac{x(B_{2}-B_{1}\theta )}{8|y|(1-\theta )}\equiv J_{a}.$$

Now, we look for the values of *x* and *y* which place the most stringent condition on the second law (Equation (24)) or, in other words, render ΔS as the least positive. This will be the worst-case scenario (WCS) in this context, as other values of *x* and *y* would yield a larger upper bound $J_{a}$. Thus, the range imposed by the WCS will hold for *all* COCs, making all of them consistent with the second law.

The first term in Equation (24) takes the minimum value if x=2. For the second term, let $y_{\min}<0$ denote the minimum value of *y*. Then, we obtain $-y_{\min}=\left[s+\left(s-1\right)+...+\left(s-\left(2s_{1}-1\right)\right)\right]=s_{1}\left(2s_{2}+1\right)$. Substituting the above values of *x* and *y* in Equation (27), we obtain the following range of *J*.

(28) $$0<J<\frac{B_{2}-B_{1}\theta }{4s_{1}\left(2s_{2}+1\right)\left(1-\theta \right)}\equiv J_{x}.$$

Therefore, it follows that for $B_{2}>B_{1}\theta$ and within the range $0<J<J_{x}$, *all* the COCs perform as an engine and satisfy the second law.

Now, from the probabilistic or average analysis, we concluded that the conditions $B_{2}>B_{1}\theta$ and the coupling range $0<J<J_{c}$ ensure the average performance as an engine. In order to compare the two ranges for *J*, note that $s_{1}\left(2s_{2}+1\right)\geq s$, where the equality is obtained for $s_{1}=1/2$ implying that, in general, $J_{x}\leq J_{c}$. This has the following important consequence. The range for the parameter *J* in which the machine behaves as an engine on average subsumes the range for *J* in which all COCs, performing as an engine, are also consistent with the second law. Conversely, if we restrict to the range $0<J<J_{x}$, allowing all COCs running as engine to follow the second law, then the average operation as an engine in that range of parameters is also consistent with the second law.

Moreover from Section 5, we learn that out of all the possible COCs with $\eta >\eta_{0}$, the maximum possible value of efficiency is obtained from Equation (26) for minimum *x*, i.e., x=2 and $|y_{min}|=s_{1}\left(2s_{2}+1\right)$, given by the following.

(29) $$\eta_{\max}=\frac{\eta_{0}}{1-\frac{4s_{1}\left(2s_{2}+1\right)J}{B_{1}}}.$$

This cycle is allowed by the second law for the condition $B_{2}>B_{1}\theta$ and in the $0<J<J_{x}$ range of coupling. Interestingly, the coupling range required for $W_{av}>0$ goes beyond $J=J_{x}$ since $J_{x}\leq J_{c}$. The case of $J_{a}=J_{c}$ is obtained when we substitute x=2,|y|=s in Equation (27), and out of all the COCs allowed in this range, the maximum efficiency is given as follows: $\eta_{0}/\left(1-4sJ/B_{1}\right)$. The latter value is same as the upper bound, $\eta_{ub}$, inferred by analysing the average performance of the system. As it can be observed, $\eta_{ub}\leq \eta_{max}$. For the special case of $\left(1/2,s_{2}\right)$ working medium, $J_{x}$ and $J_{c}$ values coincide irrespective of the value of $s_{2}$, resulting in $\eta_{max}=\eta_{ub}$.

## 6. Discussion

We have analyzed the performance of a quantum Otto engine based on a working medium with a complex energy spectrum. An insight into the possible operational regimes is hard to obtain analytically for such a system. Using a heuristic-based approach and employing techniques such as worst-case/best-case reasoning, we have highlighted a regime in which the machine definitely works as an engine on average. These set of conditions can be related to the concept of majorization for the given model. Thereby, we find that majorization serves as a more robust criterion for positive work extraction from our engine.

**Figure 5:**
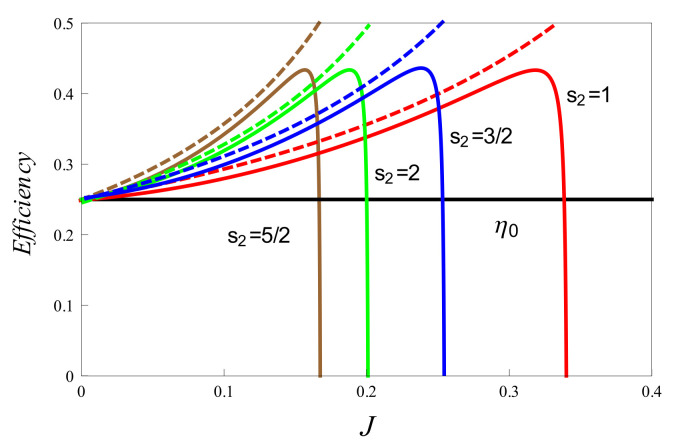
Variation of efficiency (solid lines) for different values of spin $s_{2}$, with $s_{1}=1/2,B_{1}=4,B_{2}=3,T_{1}=1$ and $T_{2}=0.5$. The corresponding upper bounds ($\eta_{ub}$) have been shown by dashed lines. The uncoupled efficiency ($\eta_{0}$) is shown by the horizontal black line.

We also introduced an analysis based on complete Otto cycles (COCs). Compared to the probabilistic analysis, the COC approach is much simpler and straightforward. The latter utilizes much less information than the ’average’ analysis, and the conclusions so obtained may not be as general. However, as a starting point, the criteria for COCs may serve as a useful heuristic to gain insight into the average performance of the Otto machine. As we have observed, there is an interesting correspondence between the COCs and the average Otto cycle with regard to the validity of the second law. One of our main results is an explicit expression for the upper bound of Otto efficiency for the coupled system. This expression reduces to the one found for the (1/2,1/2) case, with s=1 [6], or to the case of coupled, effective two-level systems [18]. The dependence of the average efficiency on coupling factor *J* and validity of the upper bound is demonstrated in Figure 5.

**Figure 6:**
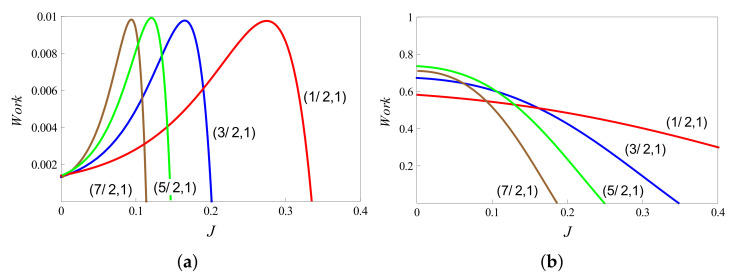
Variation of extracted work with coupling strength *J* for different spin combinations ($s_{1},s_{2}$). The fields are set at values $B_{1}=4,B_{2}=3$, and the bath temperatures are as follows: (**a**) $T_{1}=1$,$T_{2}=0.5$; (**b**) $T_{1}=6,T_{2}=3$.

**Figure 7:**
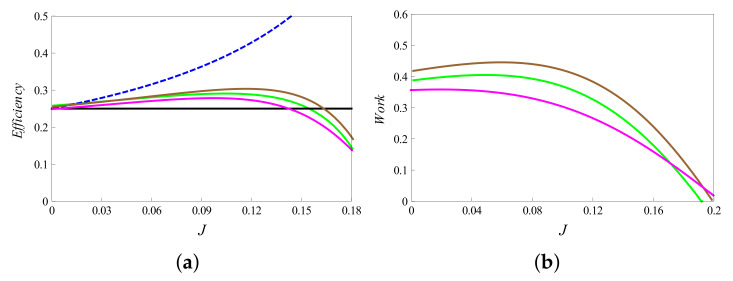
Variation of (**a**) efficiency and (**b**) work with coupling strength *J* for different spin combinations ($s_{1},s_{2}$), where s=7/2 is held fixed. The solid pink, green and brown lines show the variation for (1/2,3),(1,5/2) and (3/2,2) cases, respectively. The parameters are set at values $B_{1}=4,B_{2}=3$ and $T_{1}=4,T_{2}=2$. Here, $J_{c}=0.142$. The upper bound and the uncoupled efficiency are shown by dashed blue and black lines in (**a**), respectively. The Carnot efficiency is 0.5.

In addition to the above analytic approaches, we may also numerically study the implications of using higher spins on the performance of thermal machines. In order to make a few observations, we note that the higher “s” values shift the maximum of work to the weak coupling regimes as shown in Figure 6a. Thus, higher magnitudes of spin may be a useful resource to achieve more work output for weak coupling strengths. Numerical analysis also shows that increasing the bath temperatures may increase the work output by orders of magnitude (see Figure 6b). We also observe an extended regime of positive work extraction from the system at high temperatures and this effect is more pronounced for lower “*s*” values. Along these lines, variations of the efficiency and work output with the coupling factor *J* may be studied, where $s_{1}$ and $s_{2}$ are varied for a fixed *s* value. Figure 7 shows different cases for the case of s=7/2. Note that $\eta_{ub}$ and $J_{c}$ (which depend on *s* and not on the values of individual spins) are the same for a given *s*.

Finally, other possible domains of operation such as the refrigerator and accelerator may be addressed by using the techniques explored in this paper. The study of local thermodynamics of individual spins relative to the global performance, and other models of coupled spins featuring different interactions are some of the potential avenues of future inquiry.

## Figures and Tables

**Table 1:** Levels indicating degeneracy and energy eigenvalues ($e_{k}$) for two uncoupled spins. Here, $s_{1}<s_{2}$ with $s=s_{1}+s_{2}$ and r≡s−1/2.

*k*	$e_{k}$
1	−2sB
2,3	−2(s−1)B
4,5,6	−2(s−2)B
.	.
.	.
$(n/2-2s_{1}),...,n/2$	−2(s−r)B
$\left(n/2+1\right),...,\left(n/2+2s_{1}+1\right)$	2(s−r)B
.	.
.	.
(n−5),(n−4),(n−3)	2(s−2)B
(n−2),(n−1)	2(s−1)B
*n*	2sB

**Figure A1:**
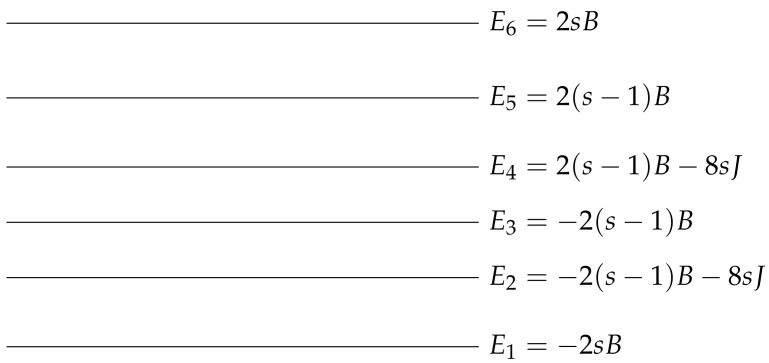
Energy levels $E_{k}$ of the coupled two-spins system (1/2,1), where s=3/2.

## Data Availability

Not applicable.

## Appendix A. PWC for the Coupled Model

The net work extracted from the system when $s_{1}$ and $s_{2}$ are uncoupled is given as $w_{av}=2\left(B_{1}-B_{2}\right)v$. Since we assume $B_{1}>B_{2}$, we need to find conditions for v>0 to hold. In other words, we have the following:

(A1) $$v=\left\{\begin{array}{c} s\left(p_{1}^{{}^{\prime }}-p_{1}+p_{n}-p_{n}^{{}^{\prime }}\right)+\\ \left(s-1\right)\left(\begin{array}{c} p_{2}^{{}^{\prime }}-p_{2}+p_{3}^{{}^{\prime }}-p_{3}+p_{n-2}-p_{n-2}^{{}^{\prime }}+p_{n-1}-p_{n-1}^{{}^{\prime }} \end{array}\right)+...+\\ \left(s-r\right)\left(\begin{array}{c} p_{n/2-2s_{1}}^{{}^{\prime }}-p_{n/2-2s_{1}}...+p_{n/2}^{{}^{\prime }}-p_{n/2}+\\ p_{n/2+1}-p_{n/2+1}^{{}^{\prime }}+...+p_{n/2+2s_{1}+1}-p_{n/2+2s_{1}+1}^{{}^{\prime }} \end{array}\right) \end{array}\right.>0,$$

where r=s−1/2. Let us denote the term in *v* with the largest coefficient *s* as follows.

$$L\equiv (p_{1}^{{}^{\prime }}-p_{1}+p_{n}-p_{n}^{{}^{\prime }})$$

We will show that L<0 implies v<0. In other words, if the term with largest coefficient in $w_{av}$ is negative, the system cannot work as an engine.

**Lemma** **A1.**
*L<0 implies v<0.*

Let us look at the explicit expression of $L\equiv \left(p_{1}^{{}^{\prime }}-p_{n}^{{}^{\prime }}\right)-\left(p_{1}-p_{n}\right)$:

(A2) $$L=\frac{e^{2sB_{2}/T_{2}}-e^{-2sB_{2}/T_{2}}}{z_{2}\left(B_{2}/T_{2}\right)}-\frac{e^{2sB_{1}/T_{1}}-e^{-2sB_{1}/T_{1}}}{z_{1}\left(B_{1}/T_{1}\right)},\;$$

where $z_{i}\left(B_{i}/T_{i}\right)$ is the partition function for the system given by Equation (5). The above expression is of the following form.

(A3) $$L=f\left(B_{2}/T_{2}\right)-f\left(B_{1}/T_{1}\right).$$

Let us observe the function $f\left(B_{1}/T_{1}\right)=p_{1}-p_{n}$. First, due to canonical form of probabilities, we know that $p_{1}>p_{n}$, and so $f(B_{1}/T_{1})>0$. Then, for a given value $B_{1}$, if we increase the temperature $T_{1}$, thereby decreasing $B_{1}/T_{1}$, we know that the difference $p_{1}-p_{n}$ decreases and vice versa. This implies that $f(B_{1}/T_{1})$ is a monotonically increasing function of $B_{1}/T_{1}$. The same is also true for $f(B_{2}/T_{2})$.

Since f(B/T)>0 is a monotonic increasing function of B/T>0, so if L<0, the following condition must hold.

(A4) $$\frac{B_{2}}{T_{2}}<\frac{B_{1}}{T_{1}}.$$

The above condition further implies $z_{2}<z_{1}$, and so we have the following.

(A5) $$p_{n}^{}-p_{n}^{{}^{\prime }}=\frac{e^{-2sB_{1}/T_{1}}}{z_{1}}-\frac{e^{-2sB_{2}/T_{2}}}{z_{2}}<0,$$

(A6) $$p_{1}^{{}^{\prime }}-p_{1}=\frac{1}{\sum_{l=0}^{2s_{}}g_{\left|s-l\right|}e^{-2lB_{2}/T_{2}}}-\frac{1}{\sum_{l=0}^{2s_{}}g_{\left|s-l\right|}e^{-2lB_{1}/T_{1}}}<0.$$

We now rewrite the expression of *v* as follows.

(A7) $$v=\left\{\begin{array}{c} s\begin{array}{c} \left[p_{1}^{{}^{\prime }}\left(1-e^{-4sB_{2}/T_{2}}\right)-p_{1}\left(1-e^{-4sB_{1}/T_{1}}\right)\right] \end{array}+\\ \left(s-1\right)\begin{array}{c} \left\{\left(p_{2}^{{}^{\prime }}+p_{3}^{{}^{\prime }}\right)\left(1-e^{-4\left(s-1\right)B_{2}/T_{2}}\right)-\left(p_{2}+p_{3}\right)\left(1-e^{-4\left(s-1\right)B_{1}/T_{1}}\right)\right] \end{array}+...+\\ \left(s-r\right)\left[\left(p_{n/2-2s_{1}}^{{}^{\prime }}+...+p_{n/2}^{{}^{\prime }}\right)\left(1-e^{-2B_{2}/T_{2}}\right)-\left(p_{n/2-2s_{1}}+...+p_{n/2}\right)\left(1-e^{-2B_{1}/T_{1}}\right)\right]. \end{array}\right.$$

Now, if L<0 and Equation (A4) holds, then in the first term above accompanying the coefficient *s*, we have the following.

$$1-e^{-4sB_{2}/T_{2}}<1-e^{-4sB_{1}/T_{1}}.$$

Similarly, in the second term of the expression for *v*, we have the following:

$$1-e^{-4\left(s-1\right)B_{2}/T_{2}}<1-e^{-4\left(s-1\right)B_{1}/T_{1}},$$

and so on until we have the following in the last term.

$$1-e^{-2B_{2}/T_{2}}<1-e^{-2B_{1}/T_{1}},$$

It is important to note that Equation (A4) does not imply any definite relation between $p_{k}$ and $p_{k}^{{}^{\prime }}$ for k=2,...,n/2. On the other hand, it is clear from Equation (A7) that $p_{k}^{{}^{\prime }}>p_{k}$ for all k=2,..,n/2 would favor the case v>0. Thus, assuming L<0, we will now consider the BCS (Best Case Scenario), mathematically written as the following:

(A8) $$p_{k}^{{}^{\prime }}>p_{k}\;\forall \;k=2,..,n/2,$$

and show that v<0. The proof is as follows.

**Proof.** It has been noted earlier that L<0 implies Equation (A4) and $z_{2}<z_{1}$. From inspection of the form of canonical probabilities, this further results in $p_{k}<p_{k}^{{}^{\prime }}$, ∀k=n/2+1,...,n. Using these relations in the normalization condition of probabilities given as $\sum_{k=1}^{n}\left(p_{k}^{{}^{\prime }}-p_{k}\right)=0,$ we have $s\sum_{k=1}^{n/2}\left(p_{k}^{{}^{\prime }}-p_{k}\right)<0$ along with the following.

$$\left(s-r\right)\left(p_{n/2+1}-p_{n/2+1}^{{}^{\prime }}\right)<0,\;...\;\left(s-1\right)\left(p_{n-1}-p_{n-1}^{{}^{\prime }}\right)<0,\;s\left(p_{n}-p_{n}^{{}^{\prime }}\right)<0.$$

Moreover, under BCS, we have the following.

$$\left(-1\right).\left(p_{2}^{{}^{\prime }}-p_{2}\right)<0,\;\left(-1\right).\left(p_{3}^{{}^{\prime }}-p_{3}\right)<0,\;...,\;\left(-r\right).\left(p_{n/2}^{{}^{\prime }}-p_{n/2}\right)<0.$$

□

Adding all the above inequalities, we arrive at the result v<0, thereby proving Lemma A1.

**Lemma** **A2.**
*L>0 implies v>0.*

Using the monotonic property of *L*, it is obvious that if L>0, the following must hold.

(A9) $$\frac{B_{2}}{T_{2}}>\frac{B_{1}}{T_{1}}.$$

It can be observed that the above condition implies $p_{1}^{{}^{\prime }}>p_{1}$ and $p_{n}>p_{n}^{{}^{\prime }}$. Equation (A9) favors v>0 as it results in the following condition:

$$1-e^{-2m_{1}B_{2}/T_{2}}>1-e^{-2m_{1}B_{1}/T_{1}},$$

in all the terms in Equation (A7), as $m_{1}>0$ for all the upper-half levels, i.e., for k=n/2+1,...,n (see Table A2). Moreover, under Equation (A9), positivity of Equation (A7) is always favored irrespective of the relation between $p_{k}$ and $p_{k}^{{}^{\prime }}$ for all k=n/2+1,...,n. As for the rest of the occupation probabilities, Equation (A9) does not imply any relation between them except for $p_{1}^{{}^{\prime }}>p_{1}$. However, it is obvious from Equation (A7) that $p_{k}^{{}^{\prime }}<p_{k}$ for all k=2,..,n/2 would *not* favor v>0.

Thus, assuming L>0, we will now consider the WCS (Worst Case Scenario), mathematically written as the following:

(A10) $$p_{k}^{{}^{\prime }}<p_{k}\;\forall \;k=2,3...,n/2,$$

and then show that v>0. This would prove *Lemma A2*.

**Proof.** The condition L>0 yields Equation (A9), resulting in $z_{2}>z_{1}$, which further implies the following.

(A11) $$p_{k}^{{}^{\prime }}<p_{k},\;k=n/2+1,...,n.$$

□

Thus, we can write the following.

(A12) $$p_{k}^{{}^{\prime }}<p_{k},\;k=2,...,n.$$

These inequalities, along with the normalization of each probability distribution, imply the following.

(A13) $$p_{1}<p_{1}^{{}^{\prime }}.$$

Now, by using Equation (A11) and the normalization of probability distributions, we can write the following:

$$s\sum_{k=1}^{n/2}\left(p_{k}^{{}^{\prime }}-p_{k}\right)>0,$$

along with the following conditions.

$$\left(s-r\right)\left(p_{n/2+1}-p_{n/2+1}^{{}^{\prime }}\right)>0,\;...,\;\left(s-1\right)\left(p_{n-1}-p_{n-1}^{{}^{\prime }}\right)>0,\;s\left(p_{n}-p_{n}^{{}^{\prime }}\right)>0.$$

Moreover, under WCS, we have the following.

$$\left(-1\right).\left(p_{2}^{{}^{\prime }}-p_{2}\right)>0,\;\left(-1\right).\left(p_{3}^{{}^{\prime }}-p_{3}\right)>0,\;...,\;\left(-r\right).\left(p_{n/2}^{{}^{\prime }}-p_{n/2}\right)>0.$$

Adding all the above inequalities, we obtain v>0, thereby proving *Lemma* 2 and concluding that L>0, or Equation (A9), is a necessary and sufficient condition for positive work extraction from the uncoupled spin system.

## Appendix B. PWC for the Coupled Model

When the spins are interacting, the work extracted is given as follows:

$$W_{av}=2\left(B_{1}-B_{2}\right)X,$$

where $X=\frac{1}{2}\sum_{k=1}^{n}m_{1}\left(P_{k}^{}-P_{k}^{{}^{\prime }}\right)$. The explicit expressions of occupation probabilities are given in Table A1. With $B_{1}>B_{2}$, we need to find the condition for which we have X>0, where the following is the case.

(A14) $$X=\left\{\begin{array}{c} s\left(P_{1}^{{}^{\prime }}-P_{1}+P_{n}-P_{n}^{{}^{\prime }}\right)+\\ \left(s-1\right)\left(\begin{array}{c} P_{2}^{{}^{\prime }}-P_{2}+P_{3}^{{}^{\prime }}-P_{3}+P_{n-2}-P_{n-2}^{{}^{\prime }}+P_{n-1}-P_{n-1}^{{}^{\prime }} \end{array}\right)+...+\\ \left(s-r\right)\left(\begin{array}{c} P_{n/2-2s_{1}}^{{}^{\prime }}-P_{n/2-2s_{1}}...+P_{n/2}^{{}^{\prime }}-P_{n/2}+\\ P_{n/2+1}-P_{n/2+1}^{{}^{\prime }}+...+P_{n/2+2s_{1}+1}-P_{n/2+2s_{1}+1}^{{}^{\prime }} \end{array}\right). \end{array}\right.$$

As shown in Appendix A, for the uncoupled spins case, the term with the largest coefficient (*s*) must be positive, i.e., L>0, for the system to run as an engine and that is possible if the system’s parameters satisfy Equation (A9). Now, we are interested in seeking additional conditions which ensure positive work extraction for the coupled case, provided that the uncoupled model works as an engine.

For completeness, we first show that the same conditions as (A9) also serve as PWC for the coupled model. In order to prove it, consider the term $L_{X}\equiv \left(P_{1}^{{}^{\prime }}-P_{1}+P_{n}-P_{n}^{{}^{\prime }}\right)$. We will first show that the opposite condition, given by Equation (A4), yields $L_{X}<0$ and so X<0. Assuming Equation (A4), we have $Z_{2}<Z_{1}$ as well as the following.

(A15) $$P_{n}^{}-P_{n}^{{}^{\prime }}=\frac{e^{-2sB_{1}/T_{1}}}{Z_{1}}-\frac{e^{-2sB_{2}/T_{2}}}{Z_{2}}<0.$$

Now, it can be observed from the explicit expressions of $P_{1}$ and $P_{1}^{{}^{\prime }}$ that for $T_{1}>T_{2}$, Equation (A4) implies $P_{1}^{{}^{\prime }}<P_{1}$. Therefore, we conclude that, under Equation (A4), $L_{X}$ is negative definite. Consider now the expression of *X*, rewritten as the following.

(A16) $$X=\left\{\begin{array}{c} s\begin{array}{c} \left[P_{1}^{{}^{\prime }}\left(1-e^{-4sB_{2}/T_{2}}\right)-P_{1}\left(1-e^{-4sB_{1}/T_{1}}\right)\right] \end{array}+\\ \left(s-1\right)\begin{array}{c} \left[\left(P_{2}^{{}^{\prime }}+P_{3}^{{}^{\prime }}\right)\left(1-e^{-4\left(s-1\right)B_{2}/T_{2}}\right)-\left(P_{2}+P_{3}\right)\left(1-e^{-4\left(s-1\right)B_{1}/T_{1}}\right)\right] \end{array}+...+\\ \left(s-r\right)\left[\left(P_{n/2-2s_{1}}^{{}^{\prime }}+...+P_{n/2}^{{}^{\prime }}\right)\left(1-e^{-2B_{2}/T_{2}}\right)-\left(P_{n/2-2s_{1}}+...+P_{n/2}\right)\left(1-e^{-2B_{1}/T_{1}}\right)\right]. \end{array}\right.$$

As it can be observed, Equation (A4) or $L_{X}<0$ implies that the following conditions:

$$1-e^{-4m_{1}B_{2}/T_{2}}<1-e^{-4m_{1}B_{1}/T_{1}}$$

hold in all the terms in Equation (A16) since $m_{1}>0$. Similar to the uncoupled case, the sign of *X* does not depend on the relation between $P_{k}$ and $P_{k}^{{}^{\prime }}$ for all k=n/2+1,...,n. However, a definite relation between $P_{k}$ and $P_{k}^{{}^{\prime }}$ for k=2,3...,n/2 is not apparent under Equation (A4). By considering the BCS that is mathematically written as follows:

(A17) $$P_{k}^{{}^{\prime }}>P_{k};\;k=2,3...,n/2$$

and then showing X<0 will prove that $L_{X}<0$ or Equation (A4) cannot make the coupled system work as an engine.

Now Equation (A17) results in the following conditions.

(A18) $$P_{k}^{{}^{\prime }}>P_{k};\;k=n/2+1,...,n-1$$

For example by using Equation (A4) and $P_{2}^{{}^{\prime }}>P_{2}^{}$ (due to Equation (A17)), we have the following.

$$P_{n-1}^{{}^{\prime }}=P_{2}^{{}^{\prime }}.e^{-4\left(s-1\right)B_{2}/T_{2}}>P_{n-1}^{}=P_{2}^{}.e^{-4\left(s-1\right)B_{1}/T_{1}}.$$

In this manner, all the relations given by Equation (A18) follow from Equations (A4) and (A17). Moreover, as noted above, Equation (A4) implies $P_{n}^{{}^{\prime }}>P_{n}^{}$. Therefore, using all these relations in the normalization condition of probabilities, we obtain the following.

$$s\sum_{k=1}^{n/2}\left(P_{k}^{}-P_{k}^{{}^{\prime }}\right)>0.$$

Relations (A18) along with $P_{n}^{{}^{\prime }}>P_{n}^{}$ imply the following.

$$\left(s-r\right)\left(P_{n/2+1}-P_{n/2+1}^{{}^{\prime }}\right)<0,...,\left(s-1\right)\left(P_{n-1}-P_{n-1}^{{}^{\prime }}\right)<0,s\left(P_{n}-P_{n}^{{}^{\prime }}\right)<0.$$

Under BCS, we have the following.

$$\left(-1\right).\left(P_{2}^{{}^{\prime }}-P_{2}\right)<0,\left(-1\right).\left(P_{3}^{{}^{\prime }}-P_{3}\right)<0,...,\left(-r\right).\left(P_{n/2}^{{}^{\prime }}-P_{n/2}\right)<0.$$

Adding all the above inequalities, we obtain the result that X<0. This means that under Equation (A4), $L_{X}$ and *X* are negative definite.

On the other hand, Equation (A9) implies $Z_{2}>Z_{1}$, which further yields $P_{n}>P_{n}^{{}^{\prime }}$. However, unlike the case with the uncoupled model, this does not determine the relative magnitudes of the ground state probabilities $P_{1}$ and $P_{1}^{{}^{\prime }}$ (explicit expressions of these probabilities are given in Table A1). Therefore, here, we cannot be sure of the sign of the quantity $L_{X}$. Now, due to Equation (A9), we note that

$$1-e^{-4m_{1}B_{2}/T_{2}}>1-e^{-4m_{1}B_{1}/T_{1}},$$

holds in all the terms in Equation (A16). Moreover, note that X>0 is always favored under this condition irrespective of the relation between $P_{k}$ and $P_{k}^{{}^{\prime }}$ for all k=n/2+1,...,n. The relation between $P_{k}$ and $P_{k}^{{}^{\prime }}$ for k=2,3...,n/2 is also not apparent under Equation (A9). As in the uncoupled case, here we will also consider the WCS written as follows.

(A19) $$P_{k}^{{}^{\prime }}<P_{k};\;k=2,3...,n/2.$$

Now, WCS results in the following conditions.

(A20) $$P_{k}^{{}^{\prime }}<P_{k};\;k=n/2+1,...,n-1.$$

For example, by using Equation (A9) and $P_{2}>P_{2}^{{}^{\prime }}$ (from (A19)), we have the following:

$$P_{n-1}^{{}^{\prime }}=P_{2}^{{}^{\prime }}.e^{-4\left(s-1\right)B_{2}/T_{2}}<P_{n-1}^{}=P_{2}^{}.e^{-4\left(s-1\right)B_{1}/T_{1}}$$

and, thus, all the relations given by Equation (A20) follow from Equations (A9) and (A19). Moreover, Equation (A9) implies $P_{n}>P_{n}^{{}^{\prime }}$, as shown above. Thus, in total, we obtain the following.

(A21) $$P_{k}^{{}^{\prime }}<P_{k}^{};\;k=2,3,4,...,n.$$

Thereby, due to the normalization condition on probabilities, we conclude the following.

(A22) $$P_{1}^{{}^{\prime }}>P_{1}.$$

**Table A1:** *Stage 1* occupation probabilities of the energy levels $E_{k}$ of the coupled spin system. $s_{1}$ is smaller of the two spins in the terms involving the factor $2s_{1}+1$.

$P_{1}=e^{2sB_{1}/T_{1}}/Z_{1}$
$P_{2}=e^{2\left(s-1\right)B_{1}/T_{1}+8sJ/T_{1}}/Z_{1}$
$P_{3}=e^{2\left(s-1\right)B_{1}/T_{1}}/Z_{1}$
$P_{4}=e^{2\left(s-2\right)B_{1}/T_{1}+8sJ/T_{1}+8J\left(s-1\right)/T_{1}}/Z_{1}$
$P_{5}=e^{2\left(s-2\right)B_{1}/T_{1}+8sJ/T_{1}}/Z_{1}$
$P_{6}=e^{2\left(s-2\right)B_{1}/T_{1}}/Z_{1}$
.
.
.
$P_{n/2-2s_{1}}=e^{B_{1}/T_{1}+8sJ/T_{1}+...+8J\left(s-\left(2s_{1}-1\right)\right)/T_{1}}/Z_{1}$
.
.
.
$P_{n/2}=e^{B_{1}/T_{1}}/Z_{1}$
$P_{n/2+1}=e^{-B_{1}/T_{1}+8sJ/T_{1}+...+8J\left(s-\left(2s_{1}-1\right)\right)/T_{1}}/Z_{1}$
.
.
.
$P_{n/2+2s_{1}+1}=e^{-B_{1}/T_{1}}/Z_{1}$
.
.
.
$P_{n-5}=e^{-2\left(s-2\right)B_{1}/T_{1}+8sJ/T_{1}+8\left(s-1\right)J/T_{1}}/Z_{1}$
$P_{n-4}=e^{-2\left(s-2\right)B_{1}/T_{1}+8sJ/T_{1}}/Z_{1}$
$P_{n-3}=e^{-2\left(s-2\right)B_{1}/T_{1}}/Z_{1}$
$P_{n-2}=e^{-2\left(s-1\right)B_{1}/T_{1}+8sJ/T_{1}}/Z_{1}$
$P_{n-1}=e^{-2\left(s-1\right)B_{1}/T_{1}}/Z_{1}$
$P_{n}=e^{-2sB_{1}/T_{1}}/Z_{1}$

In this manner, the WCS provides definite relations between the two probability distributions. We may combine Equations (A21) and (A22) to write the following.

(A23) $$\frac{P_{k}^{{}^{\prime }}}{P_{1}^{{}^{\prime }}}<\frac{P_{k}}{P_{1}}\Rightarrow \frac{e^{-E_{k}^{{}^{\prime }}/T_{2}}}{e^{2sB_{2}/T_{2}}}<\frac{e^{-E_{k}/T_{1}}}{e^{2sB_{1}/T_{1}}},\;k\neq 1.$$

Now, as shown in Appendix C, the above inequality yields the strictest condition on the permissible range of *J*, which is obtained for k=2, and is given as follows.

(A24) $$0<J<\frac{\left(B_{2}-B_{1}\theta \right)}{4s\left(1-\theta \right)}\equiv J_{c}.$$

It implies that for *J* to be in the above range, all inequalities (A23) hold good.

Now, using Equation (A20) and $P_{n}>P_{n}^{{}^{\prime }}$ in the normalization condition of probabilities, we have the following.

$$s\sum_{k=1}^{n/2}\left(P_{k}^{{}^{\prime }}-P_{k}\right)>0.$$

Equation (A21) implies the following.

$$\left(s-r\right)\left(P_{n/2+1}-P_{n/2+1}^{{}^{\prime }}\right)>0,...,\left(s-1\right)\left(P_{n-1}-P_{n-1}^{{}^{\prime }}\right)>0,s\left(P_{n}-P_{n}^{{}^{\prime }}\right)>0,$$

$$\left(-1\right).\left(P_{2}^{{}^{\prime }}-P_{2}\right),\left(-1\right).\left(P_{3}^{{}^{\prime }}-P_{3}\right),...,\left(-r\right).\left(P_{n/2}^{{}^{\prime }}-P_{n/2}\right)>0.$$

Adding all the above inequalities, we have X>0. Therefore, we conclude that, for WCS, the following conditions ensure X>0: $B_{2}>B_{1}\theta$ and $0<J<J_{c}$, where $\theta =T_{2}/T_{1}$.

Let us sum up the above discussion. There are two relevant cases.

(a) $B_{2}<B_{1}\theta$, which implies the following:
  1 $L_{X}\equiv \left(P_{1}^{{}^{\prime }}-P_{1}\right)+\left(P_{n}-P_{n}^{{}^{\prime }}\right)<0$;
  2 X<0, thereby proving that under $B_{2}<B_{1}\theta$, it is not possible for the coupled system to work as an engine at all.
(b) $B_{2}>B_{1}\theta$, which implies the following:
  1 $L_{X}$ does not bear a definite sign. Although the term ($P_{n}-P_{n}^{{}^{\prime }}$) in $L_{X}$ is positive definite, yet the sign of the other term ($P_{1}^{{}^{\prime }}-P_{1}$) is not definite;
  2 Under WCS, we are able to prove X>0 for $B_{2}>B_{1}\theta$, thereby implying that it is a necessary condition for $W_{av}>0$. However, WCS also demands $P_{1}^{{}^{\prime }}>P_{1}$ or $0<J<J_{c}$. Therefore, the latter constitutes a sufficient condition for $W_{av}>0$.
  3 When $P_{1}^{{}^{\prime }}>P_{1}$ does *not* hold, $L_{X}$ does not have definite sign. Thus, depending on the control parameters, other terms in *X* can be positive. In this case, we cannot predict the sign of $W_{av}$.

Therefore, we conclude the following regarding positive work extraction for the coupled model.

a If $L_{X}<0$ (which happens for $B_{2}<B_{1}\theta$), then $W_{av}<0$.
b If $L_{X}>0$ (which happens for $B_{2}>B_{1}\theta$ and $0<J<J_{c}$), then $W_{av}>0$.
c If no definite sign can be assigned to $L_{X}$ (which may happen even when $B_{2}>B_{1}\theta$ holds, but with no condition on the range of *J*), the system may or may not work as an engine.

## Appendix C. Condition on J from W_av_ > 0

From the conditions given by Equations (A21) and (A22), we obtained Equation (A23), which results in the following.

(A25) $$\frac{E_{k}^{}}{T_{1}}-\frac{E_{k}^{{}^{\prime }}}{T_{2}}<2s\left(\frac{B_{2}}{T_{2}}-\frac{B_{1}}{T_{1}}\right).$$

The above condition yields different possible ranges for *J* corresponding to different energies $E_{k}$. Out of these, the shortest range will clearly be permissible for *all* energy levels. Thus, we will find the strictest condition on *J* that ensures $W_{av}>0$. Let us express an energy eigenvalue as the following.

(A26) $$E_{k}^{}=m_{1}B_{1}-8m_{2}J,\;E_{k}^{{}^{\prime }}=m_{1}B_{2}-8m_{2}J.$$

As can be observed from Figure 1, there are energy bands in the spectrum of the coupled system such that the energy levels corresponding to the same band have an identical value of $m_{1}$, but different values of $m_{2}$. From the spectrum, we observe that $m_{1}$ varies from the minimum value of −2s up to 2s, while $m_{2}$ can only take positive values (see Table A2).

**Table A2:** Spin dependent factors $m_{1}$ and $m_{2}$ when the energy eigenvalues of the coupled system are expressed as the following: $E_{k}=m_{1}B-8m_{2}J$. The energy levels “*k*” which fall within the same band (i.e., having same $m_{1}$) have also been specified.

$m_{1}$	$m_{2}$	*k*
−2s	0	1
−2(s−1)	s,0	2,3
−2(s−2)	[s+(s−1)],s,0	4,5,6
−2(s−3)	[s+(s−1)+(s−2)],[s+(s−1)],s,0	7,..,10
.	.	.
.	.	.
−2(s−r)	$[s+\left(s-1\right)+...+\left(s-\left(2s_{1}-1\right)\right)],...,0$	$n/2-2s_{1},...,n/2$
2(s−r)	$[s+\left(s-1\right)+...+\left(s-\left(2s_{1}-1\right)\right)],...,0$	$n/2+1,...,n/2+2s_{1}+1$
.	.	.
.	.	.
2(s−3)	[s+(s−1)+(s−2)],[s+(s−1)],s,0	(n−9),..,(n−6)
2(s−2)	[s+(s−1)],s,0	(n−5),(n−4),(n−3)
2(s−1)	s,0	(n−2),(n−1)
2s	0	*n*

Equation (A25) now takes the following form.

(A27) $$8m_{2}\left(\frac{1}{T_{2}}-\frac{1}{T_{1}}\right)J<\left(2s+m_{1}\right)\left(\frac{B_{2}}{T_{2}}-\frac{B_{1}}{T_{1}}\right)\Rightarrow J<\frac{\left(2s+m_{1}\right)\left(B_{2}-B_{1}\theta \right)}{8m_{2}\left(1-\theta \right)}.$$

Now within one band (fixed value of $m_{1}$), it is obvious that the highest $m_{2}$ value will give the strictest condition on *J*. Now, by referring to the spectrum, we infer that for $m_{1}=-2\left(s-q\right)$ where q=0,1,2,..., the largest value of $m_{2}$ denoted by $m_{2,L}$ is

$$m_{2,L}=s+\left(s-1\right)+...+\left(s-q+1\right)=\frac{q}{2}\left(2s-q+1\right).$$

Substituting these values on R.H.S of Equation (A27), we obtain the upper limit on *J* as the following.

$$\frac{1}{2(2s-q+1)}\frac{B_{2}-B_{1}\theta }{1-\theta }.$$

Now, the strictest condition on the range of *J* will be obtained for the lowest permissible value of *q*, i.e., q=1 (since $m_{2}=0$ for q=0). Thus, we obtain the following.

(A28) $$0<J<\frac{1}{4s}.\frac{B_{2}-B_{1}\theta }{1-\theta }\equiv J_{c}.$$

Therefore, we conclude that for the above range, we have $W_{av}>0$. Note that Equation (A28) is obtained for $m_{1}=-2\left(s-1\right)$ and $m_{2}=s$, which corresponds to the first excited state of the coupled system.

## Appendix D. Proof for X > Y_1_

As discussed in the main text, for proving $\Delta S_{av}>0$ we need to show the following:

$$X>Y_{1}$$

$$\Rightarrow X-Y_{1}>0\Rightarrow X+\left(Y/s\right)>0$$

for a case where all the terms of $Y_{}$ are negative.

We will show that the PWCs, given by Equations (9) and (16), derived for the coupled model are enough to show the above relation and hence $\Delta S_{av}>0$. As already proved in the previous sections that with $B_{2}>B_{1}\theta$, the condition $J<J_{c}$ is obtained by combining the following set of conditions and then substituting k=2.

(A29) $$P_{k}^{{}^{\prime }}<P_{k}^{};\;k\geq 2$$

$$P_{1}^{{}^{\prime }}>P_{1}$$

Equation (A29) also implies maximally negative *Y*. U≡X+Y/s>0 will now be proved using Equation (A29) where *X* and *Y* are given by Equation (12).

Before starting the proof, note that all the levels contribute to *X* but only the *J* dependent levels contribute to Y/s. The steps followed for proving U>0 under relations Equation (A29) are as follows:

1. We first consider the lower half levels. With $m_{1}$ being negative for all k=1,...,n/2 (see Table A2), the total contribution from these levels to *X* takes the following form.
  $$\frac{1}{2}\sum_{k=1}^{n/2}\left|m_{1}\right|\left(P_{k}^{{}^{\prime }}-P_{k}\right)$$
  Similarly, the coefficients of these terms in Y/s can be calculated from Table A2 as $m_{2}/s$ (note that $m_{2}>0$ holds for all *k*).
  Now, we add these to obtain the coefficients of these terms in *U*, denoted by $m_{3}\equiv \frac{|m_{1}|}{2}+\frac{m_{2}}{s}$, which have been listed in Table A3. As can be observed, $m_{3}$ has a positive part given by "s" and a negative part, say $m_{4}$. The total contribution from the lower half levels to *U* is therefore written as follows.
  $$\sum_{k=1}^{n/2}\left(\frac{|m_{1}|}{2}+\frac{m_{2}}{s}\right)\left(P_{k}^{{}^{\prime }}-P_{k}\right)=\sum_{k=1}^{n/2}m_{3}\left(P_{k}^{{}^{\prime }}-P_{k}\right)$$
  $$=\sum_{k=1}^{n/2}\left(s+m_{4}\right)\left(P_{k}^{{}^{\prime }}-P_{k}\right)=s\sum_{k=1}^{n/2}\left(P_{k}^{{}^{\prime }}-P_{k}\right)+\sum_{k=1}^{n/2}m_{4}\left(P_{k}^{{}^{\prime }}-P_{k}\right)$$
  With $m_{4}<0$, the second part is positive because of Equation (A29), and the first part is considered later on.

**Table A3:** Coefficients $m_{3}$ of the terms ($P_{k}^{{}^{\prime }}-P_{k}$) in *U* with *k* varying from 1,2,...,n/2.

*k*	$m_{3}=s+m_{4}$
1	*s*
2,3	s,(s−1)
4,5,6	$\left(s-\frac{1}{s}\right),\left(s-1\right),\left(s-2\right)$
7,8,9,10	$\left(s-\frac{1}{s}-\frac{2}{s}\right),\left(s-1-\frac{1}{s}\right),\left(s-2\right),\left(s-3\right)$
.	.
.	.
$(n/2-2s_{1}),...,n/2$	$\left[s-\left(r-2s_{1}\right)-\frac{1}{s}-...-\frac{\left(2s_{1}-1\right)}{s}\right],...,\left(s-r\right)$

2. We now consider the upper half levels. The total contribution of these levels to *X* and Y/s is considered separately. The former is given as the following.
  $$\sum_{k=n/2+1}^{n}\frac{m_{1}}{2}\left(P_{k}^{}-P_{k}^{{}^{\prime }}\right)$$
  With $m_{1}$ being positive (Table A2) for all k=n/2+1,..,n, the above expression is positive because of Equation (A29).
  As for these levels’ contribution to Y/s, it is given as the following.
  $$\sum_{k=n/2+1}^{n-2}\frac{m_{2}}{s}\left(P_{k}^{{}^{\prime }}-P_{k}\right)\equiv \sum_{k=n/2+1}^{n-2}\left(m_{5}+m_{6}\right)\left(P_{k}^{{}^{\prime }}-P_{k}^{}\right)$$
  $$=\sum_{k=n/2+1}^{n-2}m_{5}\left(P_{k}^{{}^{\prime }}-P_{k}^{}\right)+\sum_{k=n/2+1}^{n-2}m_{6}\left(P_{k}^{{}^{\prime }}-P_{k}^{}\right)$$
  Note that not all the levels contribute to *Y* because many levels do not explicitly depend on *J*. Here, $m_{5}$ and $m_{6}$ are the positive and negative parts of $m_{2}/s$, respectively (see Table A4). The second part in the above equation is positive because of (A29), and the first part is considered later on.

**Table A4:** Coefficients $m_{2}/s$ (obtained from Table A2) of the terms ($P_{k}^{{}^{\prime }}-P_{k}$) in Y/s with *k* running over all upper half energy levels i.e., k=n/2+1,...,n.

*k*	$m_{2}/s=m_{5}+m_{6}$	$m_{5}$
$\left(n/2+1\right),...,\left(n/2+2s_{1}+1\right)$	$2s_{1}-\frac{1}{s}-...-\frac{\left(2s_{1}-1\right)}{s},...,0$	$2s_{1},...,0$
	.	.
	.	.
(n−9),(n−8),(n−7),(n−6)	$\left(3-\frac{1}{s}-\frac{2}{s}\right),\left(2-\frac{1}{s}\right),1,0$	3,2,1,0
(n−5),(n−4),(n−3)	$(2-\frac{1}{s}),1,0$	2,1,0
(n−2),(n−1)	1,0	1,0
*n*	0	0

3. Adding up the total contribution to *U* from all the energy levels we have the following.
  $$\sum_{k=1}^{n/2}\left(s+m_{4}\right)\left(P_{k}^{{}^{\prime }}-P_{k}\right)+\sum_{k=n/2+1}^{n}\frac{m_{1}}{2}\left(P_{k}^{}-P_{k}^{{}^{\prime }}\right)+\sum_{k=n/2+1}^{n-2}\left(m_{5}+m_{6}\right)\left(P_{k}^{{}^{\prime }}-P_{k}^{}\right)$$
  From the first and second points, we now have two parts which are yet to be proved positive. Their sum is given as follows.
  (A30) $$\sum_{k=1}^{n/2}s\left(P_{k}^{{}^{\prime }}-P_{k}\right)+\sum_{k=n/2+1}^{n-2}m_{5}\left(P_{k}^{{}^{\prime }}-P_{k}^{}\right)$$
  Using relations such as $P_{n}^{{}^{\prime }}<P_{n}$ and $P_{n-1}^{{}^{\prime }}<P_{n-1}$ (from Equation (A29)) and $P_{1}^{{}^{\prime }}>P_{1}$ in the normalization condition of the following probabilities:
  $$\sum_{k=1}^{n}\left(P_{k}^{{}^{\prime }}-P_{k}\right)=0$$
  we have the following.
  $$\sum_{k=1}^{n/2}s\left(P_{k}^{{}^{\prime }}-P_{k}\right)+\sum_{k=n/2+1}^{n-2}s\left(P_{k}^{{}^{\prime }}-P_{k}^{}\right)>0.$$
  As shown below, $m_{5}<s$. Therefore, with $P_{k}^{{}^{\prime }}<P_{k}$, we can safely replace *s* by $m_{5}$ in the above inequality, thereby proving U>0.

### Proof for ***m_5_** < **s***

In order to prove $m_{5}<s$, consider as an example the k=n−5 level, the explicit expression of the occupation probability is the following:

$$P_{n-5}=e^{-2\left(s-2\right)B_{1}/T_{1}+8sJ/T_{1}+8J\left(s-1\right)/T_{1}}/Z_{1}$$

and $m_{5}=2$ (see Table A4). On carefully observing the energy spectrum, it can be observed that the energy level corresponding to this occupation probability exists only if the sum of spins “s” in the power of the exponent satisfies 2<s. Similarly, 1<s holds in $P_{n-2}$ and 3<s holds in $P_{n-9},...,P_{n-6}$. Thus, this is true for all the energy eigenvalues. Since $\left(P_{n-5}^{{}^{\prime }}-P_{n-5}\right)<0$ (Equation (A29)), we therefore have the following.

$$s\left(P_{n-5}^{{}^{\prime }}-P_{n-5}\right)<2\left(P_{n-5}^{{}^{\prime }}-P_{n-5}\right).$$

Similarly, we have the following.

$$s\left(P_{n-2}^{{}^{\prime }}-P_{n-2}\right)<1\left(P_{n-2}^{{}^{\prime }}-P_{n-2}\right),$$

$$\begin{array}{c} s\left(P_{n-4}^{{}^{\prime }}-P_{n-4}\right)<1\left(P_{n-4}^{{}^{\prime }}-P_{n-4}\right),\\ \vdots \\ s\left(P_{n/2+1}^{{}^{\prime }}-P_{n/2+1}\right)<2s_{1}\left(P_{n/2+1}^{{}^{\prime }}-P_{n/2+1}\right). \end{array}$$

The last inequality follows from the fact $s_{1}<s_{2}$. This proves $m_{5}<s$.

$$\mathrm{Case}\;\mathrm{study}:\;s_{1}=1/2,s_{2}=1$$

As an illustration of the above proof for the upper bound of Otto efficiency, we consider the (1/2,1) coupled system (Figure A1), where n=6.

The *Stage 1* equilibrium occupation probabilities of these levels are of the form: $P_{k}=e^{-m_{1}B_{1}/T_{1}+8m_{2}J/T_{1}}/Z_{1}$, where the spin-dependent factors $m_{1}$ and $m_{2}$ have been specified in Table A5. The partition function is as follows

$$Z_{1}=\begin{array}{c} Z_{1}+2\cosh\left[2\left(s-1\right)B_{1}/T_{1}\right].e^{8sJ/T_{1}} \end{array}$$

with

$$Z_{1}\equiv 2\sum_{k=1}^{s+1/2}\cosh\left[2\left(s-k+1\right)B_{1}/T_{1}\right]=2\left(\cosh\left[2sB_{1}/T_{1}\right]+\cosh\left[2\left(s-1\right)B_{1}/T_{1}\right]\right).$$

**Table A5:** Spin dependent factors $m_{1}$ and $m_{2}$ for the (1/2,1) coupled system when the energy eigenvalues are expressed as: $E_{k}=m_{1}B-8m_{2}J$.

$m_{1}$	$m_{2}$	*k*
−2s	0	1
−2(s−1)	s,0	2,3
2(s−1)	s,0	4,5
2s	0	6

The heat absorbed from the hot bath and average works are given as follows:

$$Q_{1,av}=2B_{1}X+8JY,\;W_{av}=2\left(B_{1}-B_{2}\right)X,$$

where

$$X=\frac{1}{2}\sum_{k=1}^{n=6}m_{1}\left(P_{k}^{}-P_{k}^{{}^{\prime }}\right),\;Y=\sum_{k=2}^{(n-2)=4}m_{2}\left(P_{k}^{{}^{\prime }}-P_{k}\right)=\left(P_{2}^{{}^{\prime }}-P_{2}^{}\right)+\left(P_{4}^{{}^{\prime }}-P_{4}^{}\right).$$

**Proof.** Proof for $X>Y_{1}$ □

As discussed in the main text, we will be proving U=X+(Y/s)>0 by using Equation (A29) and the condition $P_{1}^{{}^{\prime }}>P_{1}$. Note that all levels contribute to *X* but only the *J* dependent levels ($E_{2}$ and $E_{4}$) contribute to *Y*. The steps followed for proving U>0 under relations Equation (A29) are as follows.

1. We first consider the lower half (k=1,2,3) of the levels. With $m_{1}$ being negative (see Table A5), the total contribution from these levels to *X* takes the form of the following.
  $$\frac{1}{2}\sum_{k=1}^{3}\left|m_{1}\right|\left(P_{k}^{{}^{\prime }}-P_{k}\right)=s\left(P_{1}^{{}^{\prime }}-P_{1}\right)+\left(s-1\right)\left(P_{2}^{{}^{\prime }}-P_{2}+P_{3}^{{}^{\prime }}-P_{3}\right)$$
  Similarly, contribution of lower half levels to Y/s is written as follows.
  $$Y/s=\frac{m_{2}}{s}\left(P_{2}^{{}^{\prime }}-P_{2}\right)=\left(P_{2}^{{}^{\prime }}-P_{2}\right).$$
  The total contribution of the lower half levels to *U* is as follows.
  $$U=X+Y/s=s\left(P_{1}^{{}^{\prime }}-P_{1}\right)+\left(s-1\right)\left(P_{2}^{{}^{\prime }}-P_{2}+P_{3}^{{}^{\prime }}-P_{3}\right)+\left(P_{2}^{{}^{\prime }}-P_{2}\right)$$
  $$U=s\left(P_{1}^{{}^{\prime }}-P_{1}\right)+s\left(P_{2}^{{}^{\prime }}-P_{2}\right)+\left(s-1\right)\left(P_{3}^{{}^{\prime }}-P_{3}\right)=\sum_{k=1}^{3}s\left(P_{k}^{{}^{\prime }}-P_{k}\right)+\left(-1\right)\left(P_{3}^{{}^{\prime }}-P_{3}\right)$$
  The second part is positive because of Equation (A29), and the first part is considered later on.
2. We now consider the upper half levels. The total contribution of these levels to *X* and Y/s is considered separately. The former is given as follows.
  $$\sum_{k=4}^{6}\frac{m_{1}}{2}\left(P_{k}^{}-P_{k}^{{}^{\prime }}\right)=\left(s-1\right)\left(P_{4}-P_{4}^{{}^{\prime }}+P_{5}-P_{5}^{{}^{\prime }}\right)+s\left(P_{6}^{}-P_{6}^{{}^{\prime }}\right)$$
  The above expression is positive because of Equation (A29).
  As for these levels’ contribution to Y/s, it is given as the following.
  $$Y/s=\frac{m_{2}}{s}\left(P_{4}^{{}^{\prime }}-P_{4}\right)=\left(P_{4}^{{}^{\prime }}-P_{4}\right)$$
  This part is negative and will be considered later on.
3. From points one and two the following terms in *U* are yet to be shown positive.
  $$\sum_{k=1}^{3}s\left(P_{k}^{{}^{\prime }}-P_{k}\right)+\left(P_{4}^{{}^{\prime }}-P_{4}\right)$$
  Using relations such $P_{6}^{{}^{\prime }}<P_{6}$, $P_{5}^{{}^{\prime }}<P_{5}$ (from Equation (A29)) and $P_{1}^{{}^{\prime }}>P_{1}$ in the normalization condition of probabilities, given as the following:
  $$\sum_{k=1}^{6}\left(P_{k}^{{}^{\prime }}-P_{k}\right)=0$$
  we have the following.
  $$\sum_{k=1}^{4}s\left(P_{k}^{{}^{\prime }}-P_{k}\right)>0\Rightarrow \sum_{k=1}^{3}s\left(P_{k}^{{}^{\prime }}-P_{k}\right)+s\left(P_{4}^{{}^{\prime }}-P_{4}\right)>0$$
  With $P_{4}^{{}^{\prime }}<P_{4}$ and 1<s (s=3/2 for the present case), we can safely replace *s* by 1 in the the above expression, thereby proving U>0.
